# mir-101-3p is a key regulator of tumor metabolism in triple negative breast cancer targeting AMPK

**DOI:** 10.18632/oncotarget.9072

**Published:** 2016-04-28

**Authors:** Peng Liu, Feng Ye, Xinhua Xie, Xing Li, Hailin Tang, Shuaijie Li, Xiaojia Huang, Cailu Song, Weidong Wei, Xiaoming Xie

**Affiliations:** ^1^ Department of Breast Oncology, Sun Yat-Sen University Cancer Center, Guangzhou, Guangdong, People's Republic of China; ^2^ State Key Laboratory of Oncology in South China, Sun Yat-Sen University Cancer Center, Collaborative Innovation Center for Cancer Medicine, Guangzhou, Guangdong, People's Republic of China

**Keywords:** mir-101-3p, AMPK, triple negative breast cancer, tumor metabolism

## Abstract

mir-101-3p has been reported to be a tumor suppressor and a promising therapeutic target in cancer. Recently, AMPK dysfunction has been highlighted in cancers, including breast cancer. The aim of this study is to investigate the biological roles of mir-101-3p and AMPK in breast cancer. Our research demonstrated that AMPK was up-regulated in breast cancer tissues and cell lines, especially in triple negative breast cancer (TNBC). High-expression of AMPK correlated with poor outcome in both total breast cancer and TNBC patients. Ectopic expression of AMPK improved glucose uptake, glycolysis, proliferation of TNBC cells in vitro and its tumorigenicity in vivo. AMPK was predicted to be a direct target of mir-101-3p. The luciferase reporter assay was performed to certificate this prediction. The expression of AMPK was suppressed by transfection of mir-101-3p in TNBC cells. Over-expression of mir-101-3p or knock-down of AMPK inhibited glucose metabolism and proliferation of TNBC cells in vitro. Our study provides evidence that mir-101-3p- AMPK axis could be a promising therapeutic target in TNBC targeting tumor metabolism.

## BACKGROUND

Breast cancer is the most common female malignancy worldwide, with about 240,000 new incidence per year in US [1]. Generally the diagnosis and therapy of breast cancers could depend on the TNM staging system [2] and their molecular biomarkers [3], including estrogen receptor (ER), progesterone receptor (PR), human epidermal growth factor receptor-2 (HER2) in major. Basal-like type of breast cancer (ER-, PR-, HER2- and cytokeratin 5/6+), also termed triple negative breast cancer (TNBC) in most situations, is the most aggressive subtype. TNBC patients would predict more distant metastasis, tumor recurrence, and therapy resistance [4–6]. To find efficient therapeutic targets remains an emergency for TNBC patients [7].

mir-101, with its two precursor hairpin structures transcribed from chromosome 1 and 9 separately in human [8], has been associated with carcinogenesis and cancer therapy recently [9, 10]. Dysregulation of mir-101 was reported in several malignancies, including glioma [11], liver [12], prostate [13], and breast cancer [14]. Emerging evidences have implied mir-101 to be a tumor suppressor. Mir-101 directly targets enhancer of zeste homologue 2 (EZH2), a histone methyltransferase which could promote tumor proliferation and invasion [15]. Moreover, cyclooxygenase-2 (COX-2) [16] and amyloid precursor protein (APP) [17] have been proved to be another two targets of mir-101. Recently, our research demonstrated that expression of mir-101-3p, a dominant member of mir-101, was strongly decreased in TNBC tissues and cell lines and associated with TNM stage in TNBC patients. Furthermore, mir-101-3p directly targeted MCL-1, thus inhibiting cell progression and enhancing paclitaxel sensitivity in TNBC [18].

Tumor metabolism is an old topic in the field of cancer [19]. The unique metabolism style characterized by a high rate of aerobic glycolysis plus glutaminolysis, also termed Warburg effect, provides energy and materials for cancer proliferation and invasion [20]. A series of step-control enzymes have been identified to play vital roles in regulation of Warburg effect, including hexokinase-2 (HK-2), pyruvate kinase M2 (PKM2), glucose transporter-1 (GLUT-1), and lactate dehydrogenase-1 (LDH-1) [21, 22].

Among them, AMP-activated protein kinase (AMPK or PRKA for short) has been attracting emerging focus in cancer [23]. AMPK, a heterotrimeric enzyme complex with a catalytic α-subunit and two regulatory β- and γ-subunits, is a key regulator of energy homeostasis involved in the regulation of glucose, lipid, protein and cholesterol metabolism, as well as cell cycle inhibition and apoptosis physiologically [24]. Under energy depletion, increased intracellular AMP directly binds to the regulatory γ-subunit and permits phosphorylation of the α-catalytic subunit at Thr172, thus activating AMPK. AMPK activation promotes energy generation processes, including glucose uptake, glycolysis, β-oxidation of fatty acids, etc, and inhibits energy consuming processes, such as protein or lipid synthesis [25, 26]. Recently, AMPK dysfunction has been highlighted in several malignancies [27], including breast cancer [28]. Many studies implied that AMPK is a tumor suppressor due to its linkage with LKB1 [29]. However, the roles of AMPK in cancer are still controversial. A growing number of researches suggest a duality of functions, either pro- or anti-cancer depending on context, for AMPK [30, 31]. Nonetheless, the expression and activity of AMPK in breast cancer are still unclear [28].

In this study, we investigated the expression of AMPK and its prognostic roles in breast cancer patients, predicted and further identified AMPK as a novel target of mir-101-3p in TNBC. mir-101-3p-AMPK axis could be a key regulator of tumor metabolism and progression in TNBC.

## RESULTS

### The expression of AMPK in breast cancer tissues and cell lines

**Figure 1:**
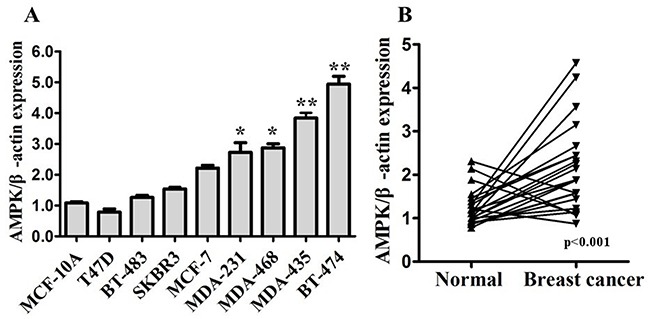
AMPK expression is up-regulated in breast cancer **A.** Expression levels of AMPK determined by western blot in human mammary cell lines, including human mammary epithelial (HME) cell lines MCF-10A, and human breast cancer cell lines. **B.** Expression levels of AMPK in 22 pairs of breast cancer tissues (Breast cancer) and their matched normal adjacent tissues (Normal). AMPK expression was normalized using β-actin expression. All of the data are shown as the means±s.e.m. * *P* < 0.05, ** *P* < 0.01. AMPK expression is up-regulated in both breast cancer cell lines and specimens.

We firstly detected expression of AMPK in human mammary cell lines (Figure 1A), including human mammary epithelial (HME) cell lines MCF-10A, and human breast cancer cell lines (MDA-MB-231, MDA-MB-435, MDA-MB-468, MCF-7, T47D, BT-474, BT-483, and SKBR3). Compared with MCF-10A, AMPK was up-regulated in BT-474 and TNBC cell lines (MDA-MB-231, MDA-MB-435, MDA-MB-468) significantly. To further evaluate the expression of AMPK in breast cancer tissues, western blot was used to detect the expression level in 22 pairs of tumor tissues and para-carcinoma (normal) tissues from breast cancer patients. The results showed that expression of AMPK in breast cancer tissues was significantly higher than in normal tissues (Figure 1B). In comparison with the normal tissues, AMPK was up-regulated in 81.8% (18/22) of the tumor samples. These results suggest that increased AMPK expression is a frequent event in human breast cancer tissues, especially in TNBC.

### The relationships between expression of AMPK and clinical parameters within breast cancer patients

**Figure 2:**
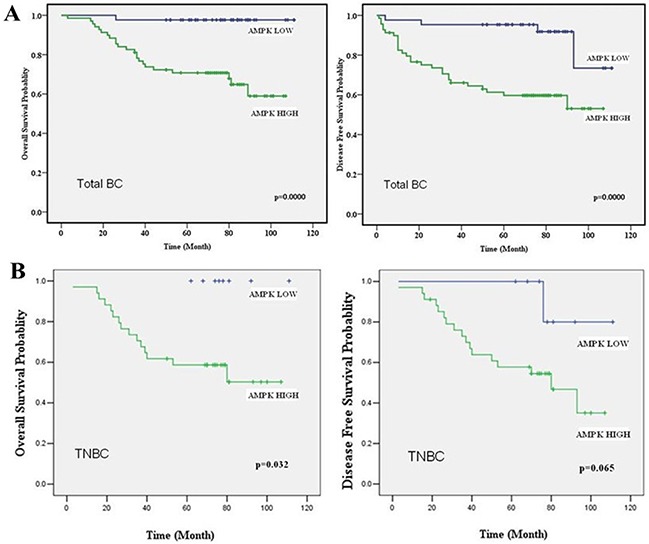
AMPK expression acts as a prognostic factor in breast cancer patients **A.** OS and DFS curves for 112 total studied patients with high or low AMPK expression. **B.** OS and DFS curves for 42 studied TNBC patients with high or low AMPK expression. High levels of AMPK correlated with shorter survival in both total breast cancer patients and TNBC patients.

We further evaluated the prognostic role of expression of AMPK in breast cancer by Immunohistochemical (IHC) staining on tissue microarray (see Supplementary Figure S1). The clinico-pathologic characteristics and AMPK expression of the breast cancer patients involved in our study are shown in Table 1. In all 112 breast cancer patients, high expression of AMPK was seen in 61.6% of total patients. High AMPK expression correlated with late TNM stage (p=0.004) and more metastasis (p<0.001). With concerns on molecular subtype, High AMPK expression was more frequent in TNBC patients (TNBC vs. Non-TNBC, 81.0% vs. 50%, p=0.001). Furthermore, markedly reduced OS and DFS were observed in the breast cancer patients who had up-regulated AMPK expression compared with the patients who exhibited low expression levels, no matter in total breast cancer patients or in TNBC patients (Figure 2). These results indicated up-regulation of AMPK could be a prognostic factor in breast cancer patients.

**Table 1:** Clinicopathological variables and MAPK expression in 112 breast cancer patients

Characteristics	Total (n=112)	MAPK low (n=43)		MAPK high (n=69)		P value
		No.	%	No.	%	
Age (years)						0.495
<50	67	24	35.8	43	64.2	
>=50	45	19	42.2	26	57.8	
Menopause						0.964
Yes	55	21	38.2	34	61.8	
No	57	22	38.6	35	61.4	
Tumor size (cm)						0.075
=<2	31	16	51.6	15	48.4	
>2	81	27	33.3	54	66.7	
LNMET						0.087
Yes	66	21	31.8	45	68.2	
No	46	22	47.8	24	52.2	
**TNM stage**						0.004*
**I-II**	**59**	**30**	**50.8**	**29**	**49.2**	
**III-IV**	**53**	**13**	**24.5**	**40**	**75.5**	
Local relapse						0.092
Yes	5	0	0	5	100.0	
No	107	43	40.2	74	59.8	
**Distant metastasis**						0.000*
**Yes**	**29**	**3**	**10.3**	**26**	**89.7**	
**No**	**83**	**40**	**48.2**	**43**	**51.8**	
ER status						0.313
Positive	84	30	35.7	54	64.3	
Negative	28	13	46.4	15	53.6	
**PR status**						0.035*
**Positive**	**46**	**23**	**50.0**	**23**	**50.0**	
**Negative**	**66**	**20**	**30.3**	**46**	**69.7**	
HER-2 status						0.127
Positive	21	5	23.8	16	76.2	
Negative	91	38	41.8	53	58.2	
P53 status						0.052
Positive	44	12	27.3	32	72.7	
Negative	68	31	45.6	37	54.4	
VEGF status						0.563
Positive	74	27	36.5	47	63.5	
Negative	38	16	42.1	22	57.9	
**TNBC status**						0.001*
**TNBC**	**42**	**8**	**19.0**	**34**	**81.0**	
**NON-TNBC**	**70**	**35**	**50.0**	**35**	**50.0**	
Ki67 status						0.520
Positive	59	21	35.6	38	0.644	
Negative	53	22	41.5	31	58.5	

* means statistically significant (*P* < 0.05).

% means percentage within the row.

TNBC, triple-negative breast cancer, means ER(—), PR(—), HER-2(—).

### Over-expression of AMPK enhanced glucose uptake and lactate production and promoted cell proliferation in TNBC in vitro and in vivo

**Figure 3:**
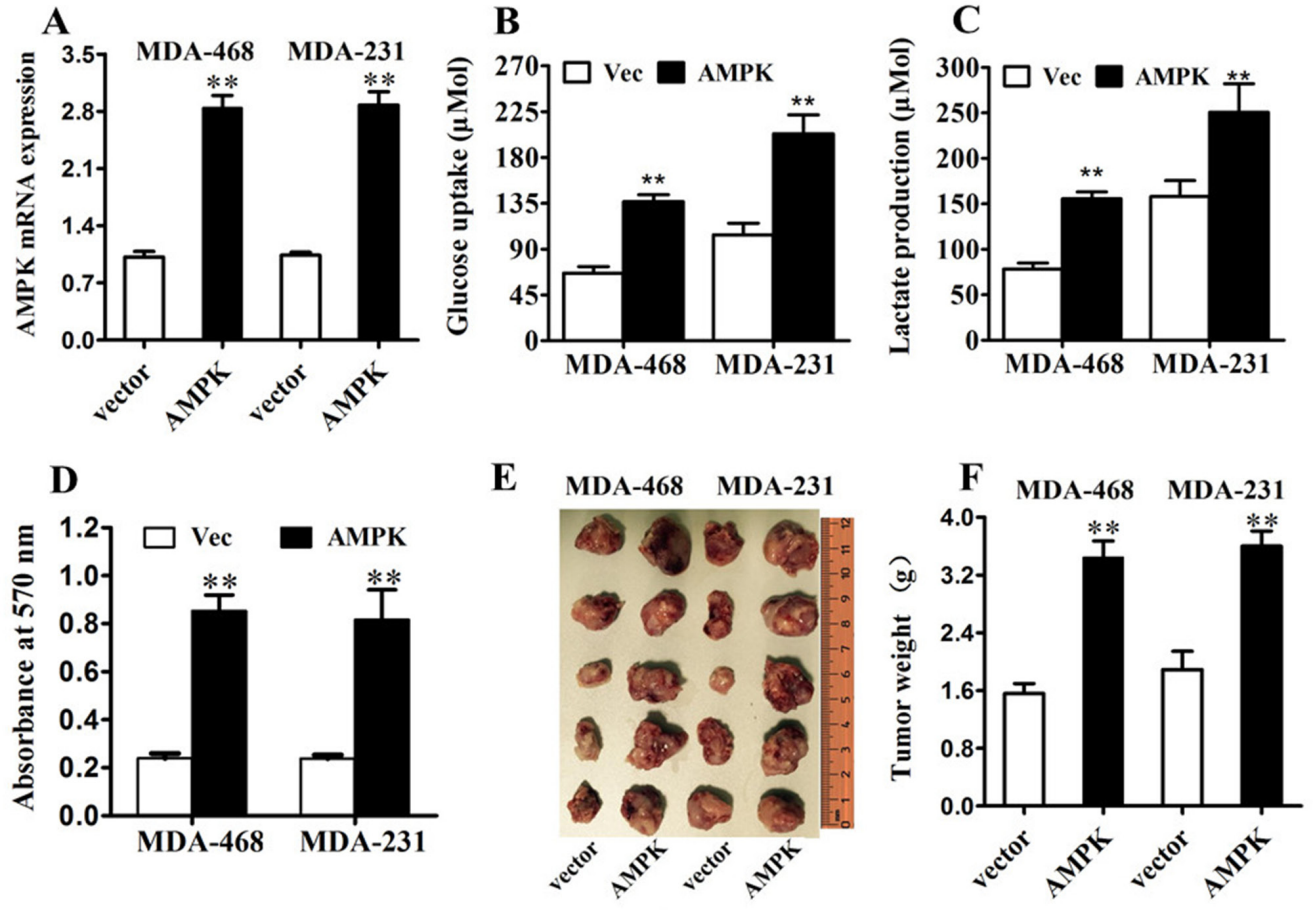
AMPK up-regulation increases glucose metabolism and proliferation in triple negative breast cancer in vitro and in vivo **A.** MDA-MB-231 and MDA-MB-468 cells were transfected with AMPK-expression vector or control vector. The transfection was successful. **B.** After the transfection, the level of glucose uptake was measured. **C.** After the transfection, the level of lactate production was measured. **D.** MTT assay was performed after the transfection. **E. & F.** Tumor growth in mouse xenograft models. MDA-MB-231 and MDA-MB-468 cells transfected with AMPK-expression vector or control vector were injected subcutaneously into BALC/c mice (five in each group). After 28 days, the mice were killed, necropsies were performed and then the tumors were weighed. All of the data are shown as the means ± s.e.m. ** *P* < 0.01.

To assess the biological effects of over-expressing AMPK in TNBC cells, ectopic expression of AMPK were transfected into MDA-MB-231 and MDA-MB-468 cells (Figure 3A). Transfection of vector-AMPK in MDA-MB-231 and MDA-MB-468 cells markedly enhanced glucose uptake and lactate production compared with vector (Figure 3B, 3C). Moreover, ectopic expression of vector-AMPK in MDA-MB-231 and MDA-MB-468 cells markedly promoted cell proliferation compared with control cells either in vitro (Figure 3D) or in vivo (Figure 3E, 3F). These results indicated that the biological effects of AMPK in TNBC cells may attribute to regulation of glucose uptake and glycolysis, thus promoting TNBC proliferations.

### mir-101-3p targeted AMPK in TNBC

**Figure 4:**
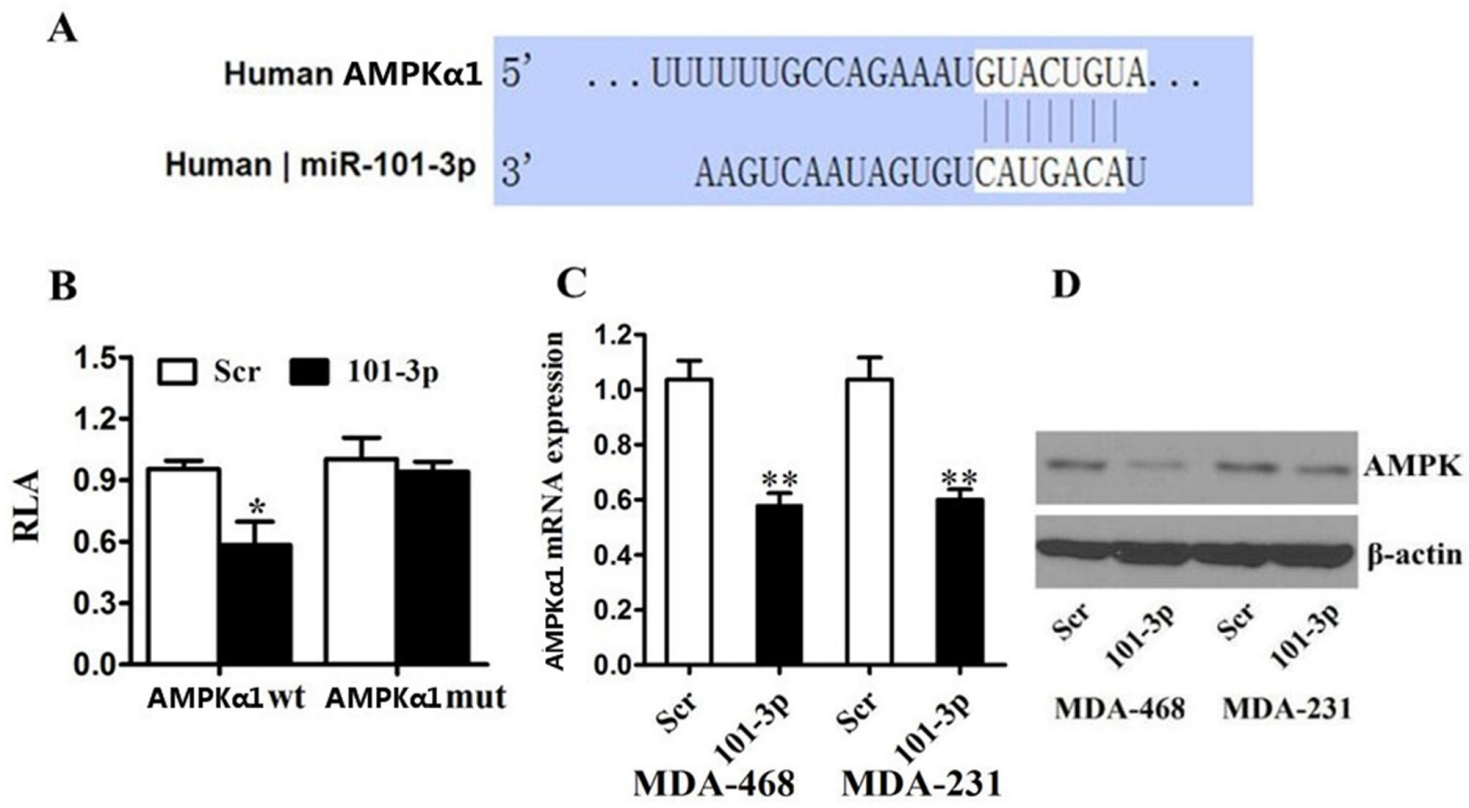
AMPK is a direct target of miR-101 in breast cancer **A.** Predicted binding between mir-101-3p and the seeds matched in the 3′-UTRs of AMPK. **B.** Luciferase assay of MDA-MB-231 cells cotransfected with mir-101-3p mimics, and a luciferase reporter containing AMPK 3′-UTR (LDHA wt) or mutant constructs (AMPK mut). **C.** MDA-MB-231 and MDA-MB-468 cells were transfected with mir-101-3p mimics or scrambled oligonucleotide. mir-101-3p overexpression inhibited the AMPK mRNA expression. **D.** MDA-MB-231 and MDA-MB-468 cells were transfected with mir-101-3p mimics or scrambled oligonucleotide. mir-101-3p overexpression inhibited the protein expression of AMPK. β-actin was used as a loading control. All of the data are shown as the means ± s.e.m. ** *P* < 0.01.

To investigate the regulation of AMPK in breast cancer, we used online softwares TargetScan to search for potential miRNAs. We found that AMPK α1-subunit (AMPKα1 or PRKAA1) was among these candidate target genes of mir-101-3p. A mir-101-3p-binding site was found in the 3′-UTR of AMPKα1 mRNA with perfect base pairing (Figure 4A). To verify whether AMPKα1 was a direct target of mir-101-3p, we subcloned the full-length 3′-UTR of AMPKα1 into the luciferase reporter vector. Addition of in vitro-produced mir-101-3p only suppressed the luciferase activity of the 3′-UTR of AMPKα1 (wild-type) upon co-transfection of the luciferase vector in MDA-MB-231 cells (Figure 4B). This inhibition was abolished when the seed sequences of the mir-101-3p target sequences were mutated in the Luc-mut vector (Figure 4B). To further confirm the effects of mir-101-3p on the expression of AMPK, we transfected mir-101-3p mimics into MDA-MB-231 and MDA-MB-468 cells and found that over-expression of mir-101-3p reduced mRNA and protein level of AMPK (Figure 4C, 4D).

### mir-101-3p-AMPK axis was a key regulator of tumor metabolism and inhibited proliferation in TNBC in vitro

**Figure 5:**
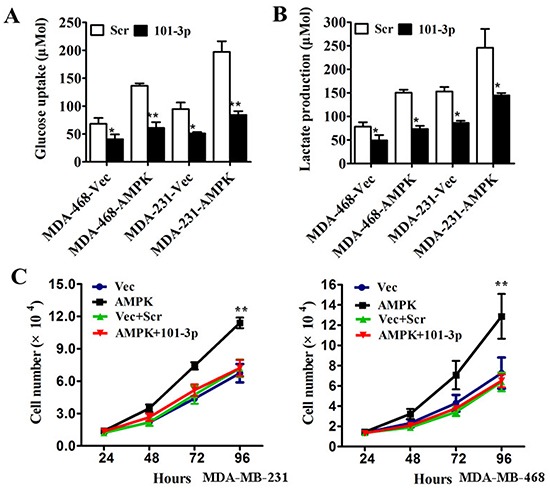
mir-101-3p-AMPK axis is a key regulator of tumor metabolism and inhibits proliferation in triple negative breast cancer in vitro **A.** MDA-MB-231 and MDA-MB-468 cells were transfected with control vector or AMPK-expression vector followed by scrambled oligonucleotide or mir-101-3p mimics. After transfection the level of glucose uptake was measured. **B.** MDA-MB-231 and MDA-MB-468 cells were transfected as described before. The level of lactate production was measured after transfection. **C.** MDA-MB-231 and MDA-MB-468 cells were transfected with control vector, AMPK-expression vector, control vector + scrambled oligonucleotide or AMPK-expression vector + mir-101-3p mimics respectively. The number of cells was counted. All of the data are shown as the means ± s.e.m. * *P* < 0.05, ** *P* < 0.01.

To assess the biological effects of over-expressing mir-101-3p in TNBC cells, vector-control, vector-AMPK, vector + scramble, or vector-AMPK + mir-101-3p mimics were transfected into MDA-MB-231 and MDA-MB-468 cells. Co-transfection of mir-101-3p and vector-AMPK in MDA-MB-231 and MDA-MB-468 cells markedly reduced glucose uptake and lactate production (Figure 5A, 5B) and attenuated cell proliferation compared with vector-AMPK (Figure 5C). These results indicated that the mir-101-3p-AMPK axis a key regulator of tumor metabolism and inhibited proliferation in TNBC in vitro, and thus mir-101-3p-AMPK axis could be a therapeutic target in TNBC.

## DISCUSSION

Although great progress have been made in the treatment and prognosis in breast cancer in the last decades, triple negative breast cancer (TNBC) is still a mystery nowadays. To find efficient therapeutic target remains an emergency for TNBC patients.

MicroRNAs (or miRNAs) play crucial roles in the post-transcriptional regulation of gene expression by binding to the 3′ untranslated region (UTR) of target mRNAs, thus inducing translation repression or degradation of mRNAs [32]. miRNAs are highly conserved and specific during evolution. To date, miRNAs has been proved to play vital roles in cancer initiation and development, acting as either tumor suppressors or oncogenes [33]. Among them, mir-101 has been reported to be a promising tumor suppressor. Our recent study has demonstrated that mir-101-3p expression was strongly decreased in TNBC tissues and cell lines and associated with TNM stage in TNBC patients. Given these favorable results, the biological functions of mir-101-3p in TNBC deserve further exploring.

The Warburg effect is believed to be the Pandora's Box of malignancies in some extent. Targeting tumor metabolism has been attracting intriguing focus in cancer therapy. Among the series of step-control enzymes in regulation of Warburg effect, AMPK is a special one. Physiologically, AMPK maintains energy homeostasis by regulation of glucose, lipid, protein and cholesterol metabolism. Under energy depletion, AMPK activation switches anabolism to catabolism in cells, inducing cell cycle arrest and even apoptosis.

However, the roles of AMPK are still controversial in cancer. Although many studies implied AMPK to be a tumor suppressor due to its upstream kinase LKB1, a growing number of researches suggest a dual role of its functions, either pro- or anti-cancer depending on context, for AMPK. Over-expression of AMPK has been addressed in several cancers, including thyroid cancer [34, 35]. It has been reported that in breast cancer, the major AMPK complex is α1β1γ1 heterotrimer [36]. Furthermore, in breast cancer, 17-β-oestradiol (E2) could directly activate AMPK through interactions of its α-subunit with estrogen receptors, implying its roles in cell proliferation [37].

In present paper, we investigated the expression of AMPK and its prognostic role in breast cancer. We showed that expression of AMPK was significantly higher in breast cancer tissues than normal tissues, especially in TNBC. Similar results were found in breast cancer cell lines. One previous study by Hadad et al. demonstrated that phosphorylated AMPK (p-AMPKα1 at Thr172) was highly expressed in normal breast epithelium and was significantly reduced in primary breast cancer samples using IHC staining [38]. However, the total AMPK expression has not been investigated in their research. Since there are multiple isoforms identified for each subunit of AMPK (two α-subunit, two β-subunits and three γ-subunits) and multiple phosphorylation sites [24], as a specific isoform, pAMPK could not represent AMPK expression in breast cancers. Thus our results are not conflicting with that of Hadad et al.

Furthermore, we analyzed the prognostic role of the expression of AMPK in breast cancer patients. We found that high expression of AMPK correlated with advanced clinical stage and more distant metastasis in breast cancer.

In our study, we demonstrated that ectopic expression of AMPK in TNBC cells markedly enhanced glucose uptake and glycolysis, the major events of Warburg effect, and promoted cell proliferation either in vitro or in vivo. Moreover, we identified AMPKα1 as a novel direct target of mir-101-3p in TNBC. Over-expression of mir-101-3p or knock-down of AMPK proteins significantly inhibited breast cancer cell proliferation and glucose metabolism.

## MATERIALS AND METHODS

### Cell lines and culture

Human mammary epithelial (HME) cell line MCF-10A, human breast cancer cell lines MDA-MB-231, MDA-MB-435, MDA-MB-468, MCF-7, T47D, BT-474, BT-483, and SKBR3 were obtained from the American Type Culture Collection (Manassas, VA, USA) and were passaged in our laboratory for less than six months after resuscitation of frozen aliquots. The breast cancer cells were cultured in Dulbecco's modified Eagle's medium (DMEM, Invitrogen, CA, USA) supplemented with 10% fetal bovine serum (FBS, GIBCO, Cappinas, Brazil), in a humidified incubator at 37°C containing 5% CO2. All cell lines were re-authenticated by short tandem repeat DNA profiling every 6 months after used.

### Western blot

Expression level of AMPK was detected in both breast cancer tissues and cell lines by Western blot. 22 pairs of tumor tissues and para-carcinoma tissues from invasive breast cancer patients diagnosed in our hospital were collected from 2015-1-1 to 2015-2-28 unintentionally. The breast cancer cell lines mentioned above were also included. Total proteins were extracted from tissues/cells and qualified with RIPA Lysis Buffer (Beyotime, Shanghai, China) and BCA Protein Assay Kit (Beyotime, Shanghai, China) according to protocols. Cell protein lysates, cytosol protein or nuclear protein was separated in 10% SDS-polyacrylamide gels, electrophoretically transferred to polyvinylidene difluoride membranes (Millipore), then detected with mouse monoclonal antibody for AMPK (PRKAA1) (SC-19126, Santa Cruz Biotechnology), mouse monoclonal antibody for β-actin (Abcam) and commercial ECL kit (Pierce). The intensity of protein fragments was quantified using Chemical DocTM XRS+ (Bio-Rad).

### Patients and specimens for tissue microarray

A total of 112 female breast cancer patients who were diagnosed by histo-pathology from October 2001 to September 2006 in Sun Yat-Sen University Cancer Center were obtained. Specimens were formalin-fixed and embedded in paraffin by standard methodology after obtained during surgery and were stored in the Department of Specimen and Resource in Sun Yat-Sen University Cancer Center. IHC of ER, PR, and HER-2 status were performed in the Pathology Department of Sun Yat-Sen University Cancer Center. All the patients included in present study did not receive any chemotherapy and radiation therapy before, and their complete clinico-pathological data, including age, histological type, lymph nodes status, tumor size, stage, local relapse, distant metastatic relapse, ER status, PR status and HER-2 status, were available and reviewed. Histological type, reclassified according to the WHO classification and stage of tumor, was based on the TNM staging system (American Joint Committee on Cancer classification). Follow-up was updated by review of records and telephone calls. The date of death and the date of relapse were used to calculate estimate overall survival (OS) and disease-free survival (DFS). Our research was permitted by our center's Ethnics Committee, and informed consents were obtained from all patients.

### Tissue microarray (TMA) construction

Representative part of the breast cancer specimens used for creating tissue microarray were selected by two experienced pathologists, using hematoxylin and eosin–stained sections which were formalin-fixed and embedded in paraffin as mentioned above. TMA block was constructed with MiniCore Control Station (ALPHELYS SARL, France) and designed by TMA Designer tissue array design software (ALPHELYS SARL, France). We used 1.0-mm core tissue biopsies and took tissues from paraffin-embedded tissue blocks to two new recipient blocks, and one core per case was arrayed. The recipient blocks were cut and placed on slides.

### Immunohistochemical (IHC) staining of AMPK and scoring system

The Labeled StreptAvidin Biotin Method was used for IHC in our study. After deparaffinizing and rehydrating, the slides were treated with 90 % methanol/3 % H_2_O_2_ solution for 15 min at room temperature to block endogenous peroxidase. Then, the slides were soaked in sodium citrate buffer (10 mM Sodium citrate, 0.05% Tween 20, pH 6.0) under 96°C for 5 min for antigen retrieval. After blocking by BSA, the following antibodies were used: mouse monoclonal antibody for AMPK (SC-19126, Santa Cruz Biotechnology). We added antibodies to the slides for overnight storage at 4°C and then incubated the slides at room temperature with biotinylated secondary antibody for 20min, and finally HRP-Streptavidin for 15min. After DAB staining, the results were graded for intensity (0-negative, 1-weak, 2-moderate, and 3-strong) and percentage of positive cells (0, 1 (1–24 %), 2 (25–49 %), 3 (50–74 %), and 4 (75–100 %)) with discrepancies resolved by consensus. The grades were multiplied to determine a score. The scores of tumors were defined as the following rule: negative (score = 0–3) and positive (score>=4) [39].

### Quantitative real-time polymerase chain reaction analysis (qRT-PCR)

For AMPK α-subunit (AMPKα1) mRNA qualification, reverse transcription and qRT-PCR reactions were performed by means of a SYBR-green-containing PCR kit (GenePharma, Shanghai, China). U6 snRNA was used as an endogenous control for miRNA detection. The expression of AMPK mRNA was quantified by measuring cycle threshold (Ct) values and normalized using the 2-^ΔΔCt^ method relative to U6 snRNA.

### Construction of luc-UTR vectors and transfection

The full-length AMPK α-subunit (AMPKα1) 3′-UTR (UUUUUUGCCAGAAAUGUACUGUA (5′→3′)) was cloned into the EcoR I and Hind III sites of the pMIR-REPORT luciferase vector (Ambion, Austin, TX, U.S.) using PCR generated fragment. A Luc-mut vector of AMPKα1 in which the first seven nucleotides complementary to the mir-101-3p seed-region were mutated by site-directed mutagenesis (Stratagene) served as a mutant control (UUUUUUGCCAGAAAUGUGCUGUA (5′→3′)). Plasmids and miRNAs were transfected into the cells at the indicated concentrations using Lipofectamine 2000 (Invitrogen, Carlsbad, CA, USA) according to the instructions.

### Luciferase assay

Luc-AMPKα1-wt and Luc-AMPKα1-mut were co-transfected separately with vitro-produced mir-101-3p intoMDA-MB-231 cells. The pMIR-REPORT β-galactosidase control vector was transfected and served as a control. Luciferase activity was measured in cell lysates 48 h after transfection using a dual-light luminescent reporter gene assay kit (Applied Biosystems). Results were normalized against β-galactosidase activity.

### MTT assay

Cell viability was examined by the 3-(4, 5-dimethylthiazol-2-yl)-2, 5-diphenyltetrazolium bromide (MTT) assay. Cells transfected with eithercontrol vector or AMPK vector were seeded at a density of 5,000 cells per well in 96-well plates and incubated at 37°C for 24 h. Cells were then incubated an additional 72 h, and the MTT assay was performed according to the manufacturer's instructions (Molecular Probes, Eugene, OR). Absorbance values were determined at 570 nm on a Spectra Max 250 spectrophotometer (Molecular Devices, Sunnyvale, CA).

### Cell growth curve

To investigate the antitumor effects of mir-101-3p in vitro, the cell growth curve was conducted. Briefly, MDA-MB-231 or MDA-MB-468 cells transfected with either vector-control, vector-AMPK, vector + scramble, or vector-AMPK + mir-101-3p mimics were seeded at a density of 5,000 cells per well in 96-well plates and incubated at 37°C for 24 h. Cells were then incubated an additional 96 h. The number of cells were imaged and counted every 24 h for drawing the Cell growth curve.

### Measurement of glucose uptake and lactate production

Either the target plasmid or miRNAs mimics were transfected into MDA-MB-231 or MDA-MB-468 cells. Cell culture media were collected after transfection for 48 h. Glucose uptake and lactate production were measured using Amplex® Red Glucose/Glucose Oxidase Assay Kit (Cat. A22189, Invitrogen) and lactate assay kit (Cat. MAK064, Sigma Aldrich) respectively. The results were normalized on the basis of total cellular protein amounts.

### Human tumor xenograft model

After vector-control or vector-AMPK was transfected into MDA-MB-231 or MDA-MB-468 cells, we used Human tumor xenograft model to investigate the biological effects of AMPK in vivo. Female BALB/c-nude mice (4–6 weeks old; Vital River Laboratories Animal, Beijing, China) were injected subcutaneously in the right fourth mammary gland with 5×106 transfected MDA-MB-231 or MDA-MB-468 cells in 100 μL of PBS with a 30-gauge needle. After 28 days, the mice were sacrificed, necropsies were performed, and the tumors were weighed. All experiments were performed in accordance with institutional guidelines and were approved by the animal care and use committee at the University of Sun Yat-Sen University Cancer Center.

### Statistical analysis

Data are presented as mean±SD from at least three separate experiments. Multiple group comparisons were performed using ANOVA with a post hoc test for subsequent individual group comparisons. The distinct expression of AMPK between tumor tissues and para-carcinoma tissues was examined by independent samples T-test. The relationships between AMPK expression and clinico-pathological parameters were examined by chi-square test. Overall survival (OS) or disease-free survival (DFS) curves were calculated by the Kaplan-Meier method and the log-rank test was used to determine the difference in OS or DFS rates between two groups. Results were considered statistically significant when *P*≤0.05 was obtained. All the statistical analyses were performed using SPSS13.0 for Windows (SPSS Inc., Chicago, IL, USA).

## CONCLUSION

In summary, our study demonstrates that AMPK could be a potential biomarker and oncogene in triple negative breast cancer. Furthermore, AMPK is identified to be a novel target regulated by mir-101-3p in TNBC. mir-101-3p-AMPK axis could be a key regulator of tumor metabolism and progression in TNBC. Our study shed novel lights in mir-101-3p and AMPK functions and TNBC research.

## SUPPLEMENTARY FIGURE


