# Supplementary figures and images for: mir-101-3p is a key regulator of tumor metabolism in triple negative breast cancer targeting AMPK
Source: Oncotarget. 2016 Apr 28;7(23):35188–98. doi: 10.18632/oncotarget.9072 (PMC5085220; doi:10.18632/oncotarget.9072)

## SUPPLEMENTARY FIGURE

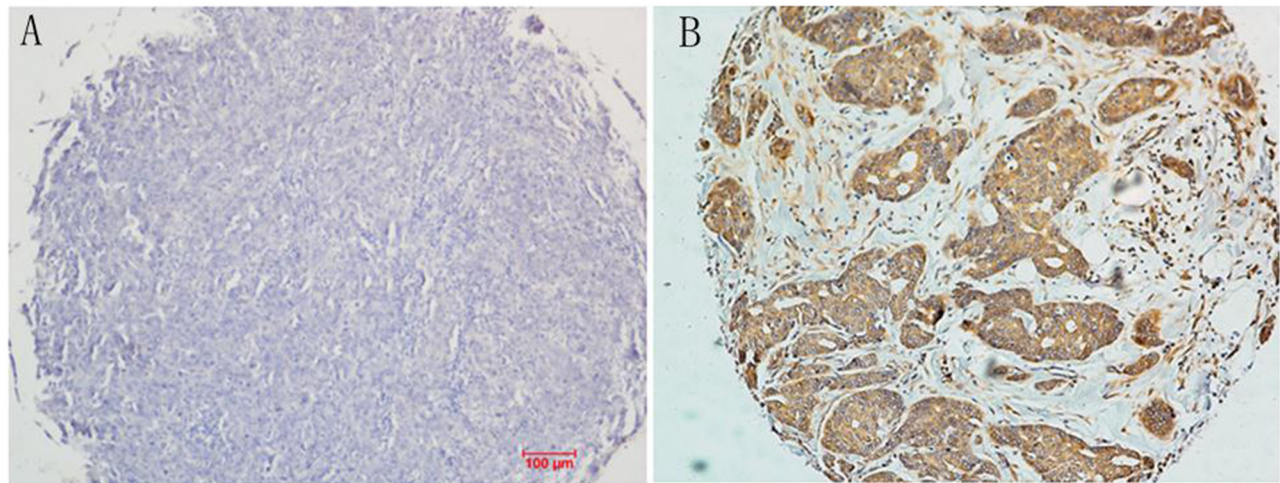

Supplementary Figure S1: IHC staining of AMPK in breast cancer.

Supplement: Supplementary file 1 [file oncotarget-07-35188-s001.pdf]
